# Blood DNA Methylation Predicts Diabetic Kidney Disease Progression in High Fat Diet-Fed Mice

**DOI:** 10.3390/nu14040785

**Published:** 2022-02-13

**Authors:** Long T. Nguyen, Benjamin P. Larkin, Rosy Wang, Alen Faiz, Carol A. Pollock, Sonia Saad

**Affiliations:** 1 Renal Research Laboratory, Kolling Institute of Medical Research, University of Sydney, Sydney, NSW 2006, Australia; Benjamin.Larkin@health.nsw.gov.au (B.P.L.); rwan5236@uni.sydney.edu.au (R.W.); carol.pollock@sydney.edu.au (C.A.P.); sonia.saad@sydney.edu.au (S.S.); 2 School of Life Sciences, Faculty of Science, University of Technology Sydney, Sydney, NSW 2007, Australia; Alen.Faiz@uts.edu.au

**Keywords:** high-fat diet, diabetes, chronic kidney disease, epigenetic, biomarker

## Abstract

Diabetic kidney disease (DKD) progresses at different rates among patients with type 2 diabetes mellitus (T2D). Early identification of patients with a higher risk of DKD progression is essential to improve prognosis. Epigenetic modifications, particularly DNA methylation, have been independently implicated in T2D and chronic kidney disease. The current study aimed to determine changes in blood DNA methylation that reflects and predicts DKD progression. C57BL/6 mice were fed a high-fat diet (HFD) from weaning and subclassified into two groups, HFD-1 and HFD-2, according to urinary kidney injury marker KIM-1/creatinine ratios (low vs. high) and histological abnormalities (mild–moderate vs. advanced). DNA methylation profiles were determined by reduced representative bisulfide sequencing (RRBS). Our results confirmed early and established DKD at week 9 and week 32, respectively. At week 32, advanced kidney injury was associated with dysregulation of methylation and demethylation enzymes in the kidney. Blood RRBS revealed 579 and 203 differentially methylated sites (DMS) between HFD-1 and HFD-2 animals at week 32 and week 9, respectively, among which 11 were common. The DMS in blood and kidney at week 32 were both related to organ development, neurogenesis, cell junction, and Wnt signalling, while the DMS in blood at week 9 suggested a specific enrichment of kidney development processes. In conclusion, our data strongly support the implication of early blood DNA methylation modifications and DKD progression in T2D that could be used to improve the disease’s prognostication.

## 1. Introduction

Diabetes is a global health concern affecting 422 million people worldwide, the majority of which have Type 2 diabetes (T2D) [1]. T2D is associated with significant comorbidities, including diabetic kidney disease (DKD), which affects 30–40% of the patient population [2,3]. DKD progression rates vary from patient to patient, even in those with similar risk factors. Although increased urinary albumin/creatinine ratio (UACR) is regarded as an early indicator of chronic kidney disease in patients with normal eGFR, it alone does not accurately predict disease progression [4]. Significant renal dysfunction can occur in almost 20% of diabetic patients with normoalbuminuria, while a proportion of patients with macroalbuminuria do not show evidence of kidney functional decline [5]. On the other hand, eGFR is regarded as a late marker of chronic kidney disease, usually occurring once significant structural damage to the kidney is evident. The mechanism for such differential progression is unclear. Identifying novel factors underlying DKD progression could improve prognostication, enabling early intervention as well as allocation of health resources and personalised therapies in patients with accelerated decline in kidney function [6].

Epigenetic modifications are mechanisms whereby different phenotypes can manifest on the same genetic blueprint, which at least in part explains why diseases progress differently among patients with similar genetic backgrounds. Changes in the pattern of DNA methylation, the most studied epigenetic mechanism, have been associated with the development of chronic kidney disease [7,8]. Patients with progressive reductions in estimated glomerular filtration rate (eGFR) have been shown to present a different whole blood DNA methylation profile compared to their counterparts with stable kidney function. The differentially methylated sites corresponded to genes involved in epithelial to mesenchymal transition (EMT), oxidative stress, and inflammation [9]. Additionally, these sites were also differentially methylated in fibrotic kidney biopsies, suggesting that similarities exist in blood and kidney DNA methylation profiles that correspond to renal fibrosis. Importantly, altered DNA methylation mediates fibroblast activation and fibrogenesis in the kidney [8], potentially indicating that modification of DNA methylation may be a strategy for the treatment of chronic kidney disease.

With respect to the role of DNA methylation in DKD, the majority of evidence to date has been in Type 1 diabetes (T1D) populations [7,10]. Although altered DNA methylation profiles have also been reported in patients with T2D [11,12], these changes are independent of co-incident DKD [12]. Essentially, there has not been a longitudinal study to identify patients with T2D at risk of DKD progression. The slow progression of DKD and the intrinsic variability in the human genome are major limitations for epigenetic investigations in humans. In addition, patients with proteinuria are uncommonly biopsied if they have T2D, which prevents correlation of epigenetic modifications in the blood with histological lesions in the kidney. Hence, in this study, we used an animal model with features consistent with T2D but with intrinsic differences in DKD progression for epigenetic profiling.

## 2. Materials and Methods

### 2.1. Animals

The study was approved by the Animal Ethics Committee of the Northern Sydney Local Health District (RESP/15/22 and RESP/18/108). All studies were performed in accordance with the relevant guidelines and regulations in the Australian Code of Practice for the Care and Use of Animals for Scientific Purposes. C57BL/6 mice (3 weeks) were fed a high-fat diet (HFD, 20 kJ/g, 43.5% calorie as fat, Specialty Feed, Glen Forrest, WA, Australia) or standard rodent chow (11 kJ/g, 14% calorie as fat, Gordon’s Speciality Stockfeeds, Yanderra, NSW, Australia). We have previously demonstrated that animals fed a HFD demonstrate glucose intolerance and insulin resistance from 9 weeks [13]. The HOMA-IR score, which represents insulin resistance, was calculated as reported previously [13,14]. Animals were kept on the same diet until sacrifice at week 32, at which timepoint animals fed a HFD demonstrated renal oxidative stress, inflammation, interstitial fibrosis, and dysfunction [15], albeit with significant animal to animal variation. A total of 11 mice were fed chow and 21 mice were fed HFD. Blood was collected into EDTA collection tubes from the heart during anaesthesia at endpoints. Plasma was isolated in supernatant following centrifuging the blood at 1500× *g* for 10 min. For a subset of week 32 animals, blood samples were also collected at week 9 from tail vein for DNA methylation analysis. A 24-h urine sample was collected 2 days before cull using metabolic cages. Blood and urine samples were stored at −20 °C until analysis.

### 2.2. Group Classification

The pathologic paradigm for kidney disease progression is advancing tubular injury and interstitial fibrosis [16]. The addition of renal tubule-specific biomarkers such as Kidney injury marker (KIM)-1 and neutrophil gelatinase-associated lipocalin (NGAL) have been shown to significantly improve prognostication [17,18,19]. Using a proteomic approach, recent studies have validated KIM-1 as one of the most significant biomarkers for predicting diabetic nephropathy and future cardiovascular events in T2D patients [20,21]. As such, in this study, HFD-fed animals were grouped into two groups HFD-1 and HFD-2 according to urinary KIM-1/creatinine ratio (UKCR). Animals in the highest tertile for UKCR were considered “advanced” or “fast progressors” (HFD-2), while the rest were considered to have mild–moderate kidney injury (HFD-1). Renal histopathology was then used for validation of DKD stages.

### 2.3. Blood and Urine Assay

The plasma levels of triglyceride (TG) were measured using Roche triglyceride reagent GPO-PAP (Roche Life Science) and the levels of non-esterified fatty acid (NEFA) were measured using Wako HR Series NEFA-HR(2) kit (Fujifilm, Tokyo, Japan). Blood insulin and urinary albumin concentration were measured using Ultra-Sensitive Mouse Insulin ELISA and Mouse Albumin ELISA Kits (CrystalChem, Elk Grove Village, IL, USA), respectively. Urinary KIM-1 was measured using a Mouse KIM-1 ELISA Kit (Abcam, Cambridge, UK). The concentrations of urinary and blood creatinine were determined using Urinary and Blood Creatinine Assays, respectively (Cayman Chemicals, Ann Arbor, MI, USA). All assays were performed according to the manufacturers’ instructions.

### 2.4. RNA Extraction and Quantitative Real-Time PCR

Total RNA of kidney tissues was isolated using RNeasy Plus Mini Kit (Qiagen Pty Ltd., CA, USA) according to the manufacturer’s instructions. The purified total RNA was used as a template to generate the first-strand cDNA using the First Strand cDNA Synthesis Kit (Roche Life Science, North Ryde, NSW, Australia). The amplicons of target genes were amplified using KiCqStart^®^ SYBR^®^ Green Primers Predesigned primers (Sigma-Aldrich, North Ryde, NSW, Australia). Gene expression was standardized to β-actin mRNA and log-transformed. Primers’ sequences are as previously published [22].

### 2.5. Immunoblot

Proteins (20 μg) were electrophoresed and electroblotted onto Hybond nitrocellulose membranes (Amersham Pharmacia Biotech, Amersham, UK), which were then incubated with a primary antibody at 4◦C overnight. The primary antibodies used for immunoblotting included manganese superoxide dismutase (SOD2, 1:2000, EMD Millipore, NSW, Australia) and KIM-1 (ab119596, dilution 1:100, Abcam, Cambridge, UK). α-Tub (T5168, 1:5000, Sigma-Aldrich, St. Louis, MO, USA) was used as the housekeeping protein. Subsequently, the membrane was incubated with a horseradish peroxidase-conjugated secondary antibody (1:5000, Cell Signalling, Danvers, MA, USA). The immunoblots were developed by adding Luminata Western HRP Substrates (Millipore, Burlington, MA, USA) to the membrane and exposed for an appropriate duration using ImageQuant LAS 4000 (Fujifilm, Tokyo, Japan). ImageJ (National Institutes of Health, Bethesda, MD, USA) was used for densitometric analyses.

### 2.6. Reduced Representative Bisulfide Sequencing (RRBS)

DNA was extracted from the blood and kidney of the same animals using a DNeasy Blood and Tissue Kit (Qiagen, Hilden, Germany). Reduced Representation Bisulfite Sequencing (RRBS) DNA library preparation was performed by the Australian Genome Research Facility (AGRF, Melbourne, Australia). The RRBS library was produced using the NuGEN Ovation^®^ RRBS Methyl-Seq system. Reads (30 million, 100 bp single-read) were assessed using FastQC v.0.11.8 [23], then trimmed using Trim Galore v0.5.0 [24]. Further trimming was performed to remove NuGEN’s RRBS specific primer. Bismark v0.21.0 [25] allowed mapping to GRCm38 builds for the mouse genome. Alignments were performed using Bowtie 2 v2.3.4 [26], with default parameters allowing 0 mismatch in a 20 bp seed. Alignments were then deduplicated using a barcoded RRBS mode of Bismark. Library sizes were limited to loci with sequencing coverage (the total read counts of both methylated and un-methylated intensity) ≥ 8. The M value was computed using the formula: M = log2((me + α/un + α)) with α = 0.1 as an offset to avoid log transformation of Zero or infinitive values. It is important to note that the sequencing depth of RRBS was much lower than microarray-based assay, hence the smaller α [27]. *p*-values and adjusted *p*-values (False Discovery Rate, FDR) of each pair-wise comparison were calculated based on M-values using the limma package [28]. Beta values, which reflect methylation levels, were converted from M-values as per previously described [29], which were used for data visualisation. A CpG site was deemed to be differentially methylated if FDR was less than 0.05 and the difference in methylation levels (deltaB) was greater than 0.05. Finally, differentially methylated genes were submitted to g:Profiler (https://biit.cs.ut.ee/gprofiler/gost, accessed on 8 August 2021) for Gene set enrichment analysis. Blood DNA methylation profiling was undertaken on the same animals at week 9 and week 32 and common differentially methylated CpGs were detected. Pathway enrichment in blood at week 32 was correlated with that in the kidney.

### 2.7. Histology

Formalin-fixed kidneys were sectioned at 4 μm thickness and stained for Hematoxylin and Eosin (H&E) and Pico-Sirius Red (PSR). The slides were assessed using a light microscope (Leica, Weztlar, Germany). The degree of glomerulosclerosis was scored in twenty glomeruli per slide in a blinded manner. PSR images were used to score glomerular and tubular injury, as well as to estimate interstitial fibrosis by Image J software (National Institutes of Health, Bethesda, MD, USA).

### 2.8. Statistical Analysis

Unpaired *t*-tests and one-way ANOVA followed by Fishers Least Significant Difference (LSD) posthoc test were used for statistical analysis. Data are presented as mean ± SEM. *p* < 0.05 was considered significant.

## 3. Results

### 3.1. High-Fat Diet Induced Diabetic Kidney Disease

As previously shown, mice fed the HFD from weaning to 9 weeks of age had increased body weight and fat mass (*p* < 0.001, Supplementary Figure S1A). The levels of triglyceride and NEFA were also higher in these mice in comparison to chow-fed animals (Supplementary Figure S1B). Glucose intolerance was significantly higher (*p* < 0.001), while insulin resistance index was also significantly elevated (*p* < 0.05) (Supplementary Figure S1C), suggesting T2D.

**Figure 1:**
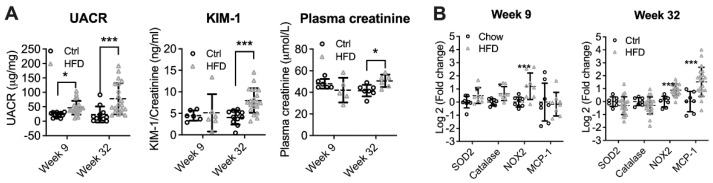
The development and progression of DKD in high-fat diet (HFD) fed animals at week 9 and week 32. (**A**) Urinary albumin/creatinine ratio (UACR), Kidney injury marker (KIM)-1 to creatinine ratio, and plasma creatinine. (**B**) Renal mRNA expression of oxidative stress and inflammation markers. * *p* < 0.05, *** *p* < 0.001 vs. Ctrl.

At week 9, UACR was significantly increased in HFD-fed mice (*p* < 0.05, Figure 1A). However, urinary KIM-1/creatinine ratio (UKCR) and plasma creatinine were unchanged, suggesting early DKD. At week 32, in addition to albuminuria (*p* < 0.001), UKCR and plasma creatinine were also significantly elevated (*p* < 0.001 and *p* < 0.05, respectively). At week 9, the mRNA expression of the oxidative stress marker NAPDH oxidase (NOX)2 was significantly increased (*p* < 0.001). This was coupled with higher mRNA expression of antioxidant enzymes including superoxide oxidase (SOD2, *p* < 0.05) and catalase (*p* < 0.05). No change was found in the expression of inflammatory markers monocyte chemoattractant protein (MCP)-1 and tumour necrosis factor (TNF)α (Figure 1B). In contrast to week 9, the renal expression of superoxide dismutase (SOD)2 and Catalase in HFD-fed animals showed trends to reduction at week 32, while NAPDH oxidase (NOX)2 remained increased (*p* < 0.001). MCP1 was also significantly elevated at week 32, suggesting renal inflammation (*p* < 0.001).

### 3.2. DKD Progression Rates Vary among HFD-Fed Mice

**Figure 2:**
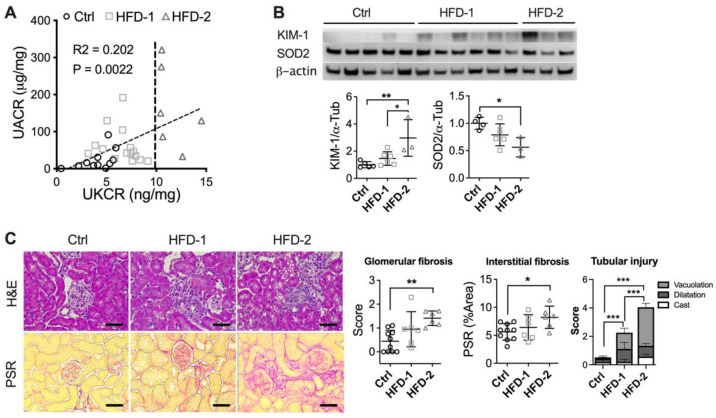
DKD progression rates vary among HFD-fed mice. (**A**) Urinary albumin/creatinine ratio (UACR) and urinary KIM-1/creatinine ratio (UKCR); (**B**) Renal protein expression of KIM-1 and antioxidant enzyme SOD2 (**C**) Histological staining: Hematoxylin and Eosin (H&E) and Pico-Sirius Red (PSR). * *p* < 0.05, ** *p* < 0.01, *** *p* < 0.001. HFD-2: mice with advanced kidney injury, HFD-1: mice with mild–moderate kidney injury.

As described in the methods section, HFD-fed animals were then subclassified into HFD-2 and HFD-1 based on UKCR. UKCR correlated positively with UACR (*p* < 0.001, Figure 2A) and was also consistent with the protein expression of KIM-1 in the kidney, where HFD-2 animals showed a higher level of renal KIM-1 compared to either control (*p* < 0.01) or HFD-1 (*p* < 0.05) (Figure 2B). In parallel, renal protein expression of SOD2 was decreased (*p* < 0.05, Figure 2B), while 8-hydroxy-2’ -deoxyguanosine (8-OHdG) was increased in HFD-2 animals in comparison to the control (*p* < 0.05, Supplementary Figure S2), suggesting a high level of oxidative stress. In comparison, no significant change was found in the kidney of HFD-1 mice. Importantly, HFD-2 mice showed significant increases in glomerular (*p* < 0.05) and tubulointerstitial fibrosis (*p* < 0.05) in comparison to the control (Figure 2C). Tubular injuries including tubular vacuolation and cast formation were also more substantial in HFD-2 group (*p* < 0.001). In comparison, the kidney of HFD-1 mice showed no significant changes in glomerular and interstitial fibrosis and less severe tubular injury, confirming milder DKD.

### 3.3. Renal DNA Methylation Was Impaired in Mice with Advanced DKD

**Figure 3:**
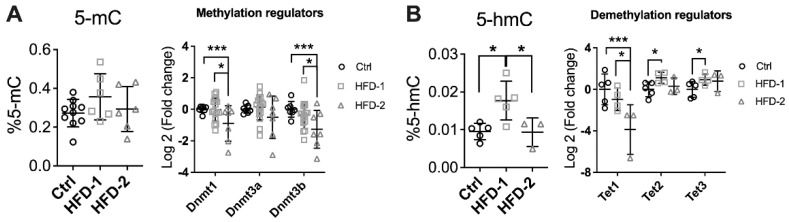
Renal DNA methylation was impaired in mice with advanced DKD. (**A**) Percentage of 5-methylcytosine (5-mC) and mRNA expression of DNA methyltransferases (DNMT), (**B**) Percentage of 5-hydroxylmethylcytosine (5-hmC) and renal mRNA expression of demethylation enzymes (ten-eleven translocation, TET). * *p* < 0.05 and *** *p* < 0.001. HFD-2: mice with advanced kidney injury, HFD-1: mice with mild–moderate kidney injury.

Body weight, fat mass, blood lipid, and glucose tolerance were similar between HFD-2 and HFD-1 mice despite different DKD stages at week 32 (Figure S3), thus excluding differential metabolic parameters as a cause of differential renal progression and pathology. Hence, epigenetic changes were explored. DNA methylation is the most stable form of epigenetic modification, involving the transfer of a methyl group onto the C5 position of the cytosine to form 5-methylcytosine, which can occur passively or is mediated by DNA methyltransferases (DNMTs) [30]. At week 32, although the level of 5-methylcytosine (5-mC) in the kidney was not different among groups, the mRNA expression of DNMT 1 and 3b was significantly downregulated in the kidney of HFD-2 mice compared to both the control and HFD-1 (*p* < 0.001 and *p* < 0.05, respectively, Figure 3A). DNMT3a also showed a trend to decrease.

**Figure 4:**
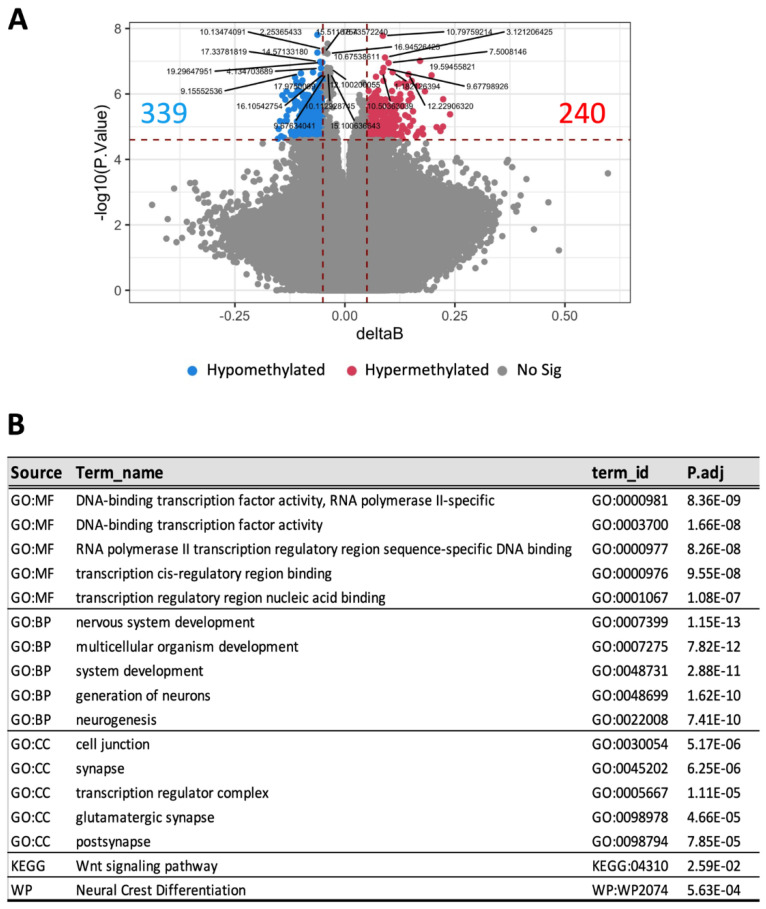
Comparison of blood DNA methylation profiles between HFD-fed mice with advanced versus mild DKD at week 32. (**A**) Volcano plots of differentially methylated CpGs, (**B**) Gene-get enrichment analysis. GO: Gene annotation, MF: Molecular function, BP: Biological process, CC: cellular compartment, KEGG: Kyoto Encyclopedia of Genes and Genomes, WP: Wikipathways. *n* = 3.

On the other hand, the renal level of 5-hydromethylcytosine (5-hmC), a by-product of DNA demethylation, was significantly increased in HFD-1 group (*p* < 0.05, Figure 3B) in comparison to the control and HFD-2 animals (*p* < 0.05, Figure 4B). The mRNA expression of TET1, the first member of the Ten-eleven translocation methylcytosine dioxygenase (TET) family, which catalyses the oxidation of 5-mC to 5-hmC, was unchanged in HFD-1 but significantly suppressed in HFD-2 animals (*p* < 0.05). In contrast, TET2 and TET3 were upregulated only in the kidney of HFD-1 group (*p* < 0.05 and *p* < 0.05, respectively). Overall, the data suggest that DNA methylation machinery was differentially modulated depending on the level of kidney injury. Consistently, kidney DNA methylation profiling by RRBS suggests an enrichment of genes involved in the TGF-β signalling pathway, which is well-known as a key mediator of tissue fibrosis (Supplementary Figure S4B).

### 3.4. Different Blood DNA Methylation Profiles between Mice with Advanced and Mild DKD

**Figure 5:**
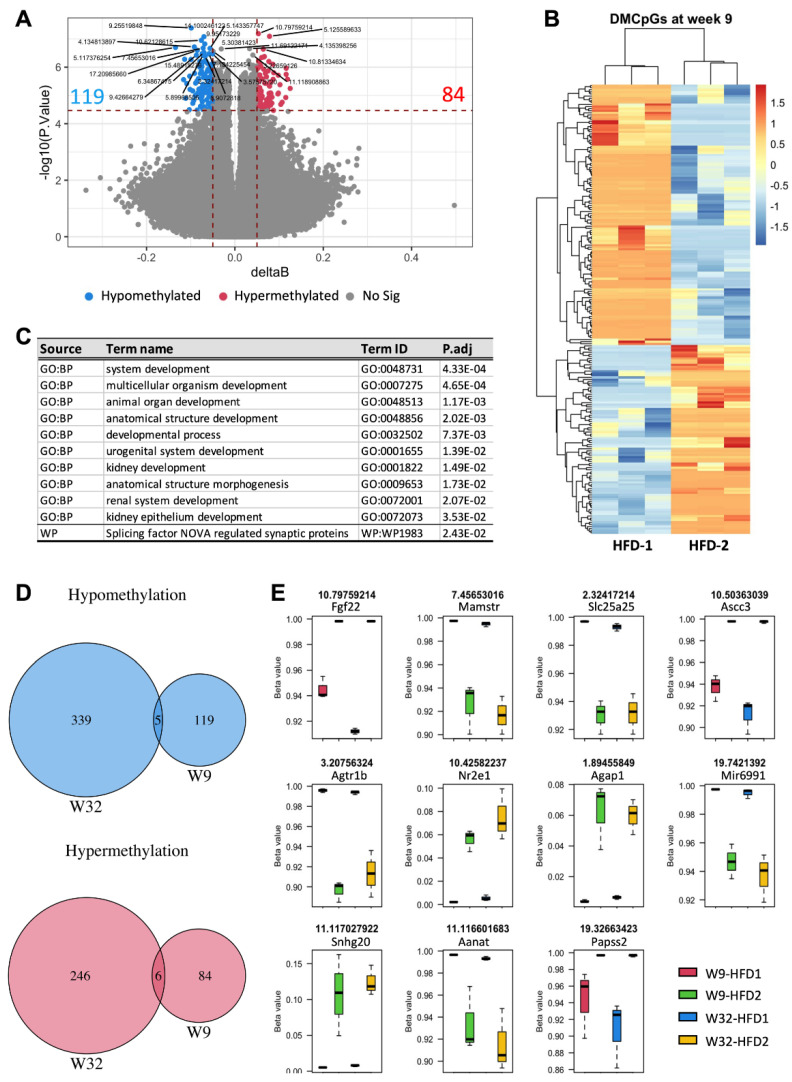
Comparison of blood DNA methylation profiles at week 9 between HFD-fed mice with advanced and mild DKD by week 32. (**A**) Volcano plot, (**B**) Heatmap of differentially methylated CpGs (DMCpGs), (**C**) Gene set enrichment analysis, (**D**,**E**) Common hypomethylated and hypermethylated CpGs in the blood of HFD-2 vs. HFD-1 group at week 9 (W9) and week 32 (W32). GO: Gene annotation, BP: Biological process, WP: Wikipathways. *n* = 3.

Altered blood DNA methylation patterns have been linked to the development of DKD [8]. In our study, blood DNA methylation profiling identified 339 hypomethylated and 240 hypermethylated loci in HFD-fed animals with advanced DKD compared to those with a milder phenotype (Figure 5A). Multiple molecular functions, biological processes, and signalling pathways were shown to be significantly enriched according to gene-set enrichment analysis (Figure 5B). These include transcriptional factors, neuronal development, cell junction, and Wnt signalling pathway. Importantly, similar processes were also enriched in the kidney of the same animals (Supplementary Figure S4B), suggesting that changes in blood DNA methylomes were reflective of kidney-specific epigenetic changes during DKD progression.

### 3.5. Blood DNA Methylation Markers Were Detectable in Early DKD

We then addressed whether these changes were also detectable in the early phase of diabetes mellitus. Blood DNA methylation profiling in animals at week 9 identified 119 hypomethylated and 84 hypermethylated CpGs in animals that progressed to advanced DKD by week 32. Gene-set analysis suggests the enrichment of multiple biological processes, all of which were related to developmental pathways (Figure 5C). Strikingly, four in the top ten significant terms were kidney-relevant, further supporting the implication of epigenetic modifications in blood and renal pathophysiology.

Among the significant differentially methylated CpGs in the blood at week 9, eleven CpGs including five hypomethylated and six hypermethylated were also evident at week 32 (Figure 5D,E). The hypomethylated CpGs included solute carrier family 25 member 25 (Slc25a25), arylalkylamine N-acetyltransferase (Aanat), microRNA 6991 (Mir6991), angiotensin II receptor, type 1b (Agtr1b), and MEF2 activating motif and SAP domain containing transcriptional regulator (Mamstr) (Figure 5E). The hypermethylated CpGs were fibroblast growth factor 22 (Fgf22), activating signal cointegrator 1 complex subunit 3 (Ascc3), 3′-phosphoadenosine 5′-phosphosulfate synthase 2(Papss2), small nucleolar RNA host gene 20(Snhg20), nuclear receptor subfamily 2, group E, member 1 (Nr2e1) and ArfGAP with GTPase domain, ankyrin repeat, and PH domain 1 (Agap1).

## 4. Discussion

It is unclear why the development and progression of DKD vary among patients. In this study, using an animal model of HFD-induced DKD, we demonstrated that DNA methylation and demethylation enzymes were dysregulated in the kidneys of mice with progressive kidney injury. The blood DNA methylation pattern was different in these animals compared to those with a mild–moderate phenotype. Common biological processes and signalling pathways in blood and kidney were enriched at the endpoint; DNA methylation changes in the blood at an earlier phase were proved to be relevant to kidney development processes. Importantly, eleven common differentially methylated genes were detected in the blood at both timepoints, suggesting that these specific changes occurred early and persisted over time, which could be used as a marker to predict DKD progression.

The development and progression of DKD in T2D patients are considered to be multifactorial and polygenic. Gene-modified or chemically induced animal models of T2D tend to manifest advanced DKD in the majority of if not in all animals, and are therefore not suitable for studying heterogenicity in DKD progression. Our model relied solely on HFD to induce obesity and T2D, an insult that closely resembles the most common aetiology of T2D in human. Importantly, the dietary stimulus is gradual so that heterogenicity among animals regarding DKD development and progression accumulates.

Consistent with our previous studies [31], mice fed a HFD from week 3 to week 9 were obese, diabetic, and albuminuric. However, no significant changes in markers of tubular injury, kidney function, as well as renal oxidative stress and inflammation were evident at this timepoint, suggesting that week 9 corresponded to an early phase of DKD. By week 32, histopathological features of DKD were evident, indicating established DKD. UKCR showed strong correlations with intrarenal KIM-1, renal oxidative stress, and structural abnormalities at week 32, suggesting that this was a reliable marker to classify the stage of DKD progression, and this is consistent with human studies in which KIM-1 predicts early kidney functional decline as well as end-stage kidney disease [20,21,32].

Given the same genome and environmental conditions, the differences in renal pathophysiology reflect differences in how the genome reacted to the environment. Hence, epigenetic changes were implicated. Epigenetic modifications, particularly DNA methylation, have been associated with renal function decline in patients with diabetic kidney disease in both T1D [7] and T2D populations [33]. During DNA methylation, a methyl group is transferred onto C5 position of cytosine to form 5-methylcytosine (5-mC). This process is mediated by DNMTs with DMNT1 functioning as the maintenance methyltransferase and DMNT3a/b functioning as “de novo” methyltransferases [30]. DNA methylation is stable but can be reversed following a DNA demethylation cascade, of which the first step of hydroxylation (5-mC to 5-hmC) is catalysed by TETs [34]. The balance between the two enzyme families orchestrates the methylome and transcriptome during cellular replication and differentiation. In our study, the expression of these enzymes was significantly altered in the kidney of animals with progressive CKD, while it maintained relatively balanced in the kidney of those with non-progressive CKD. Such impairments of the renal DNA methylation machinery may underline the higher level of DKD progression in certain T2D cohorts. Indeed, multiple studies have shown altered expression of DNMTs and TETs in kidney diseases. DNMT1 deficiency has been demonstrated to induce global DNA hypomethylation in nephron progenitor cells, leading to downregulation of genes crucial for initiation of nephrogenesis such as Wt1 (proximal nephron marker) and its target Wnt Family Member 4 (Wnt4) [35]. Regarding renal fibrosis, the roles of DNMTs are unclear. However, previous studies have shown both positive and negative effects of DNMTs in cardiac fibrosis [36]. Similar to DNMTs, the roles of TETs in renal fibrosis have not been elucidated, while both up- and down-regulation have been reported in other fibrotic diseases [36].

As kidney and blood are constituted of very different cell types, their DNA methylation profiles are intrinsically different. As such, focusing on individual differentially methylated sites that were common between kidney and blood could lead to neglection of important findings. In this study, although kidney methylation profile was investigated in a small number of animals, by comparing kidney and blood DNA methylation profiles at a pathway level, we found strong similarities between the two methylomes in association with advanced kidney injury, suggesting an interesting epigenetic correlation between kidney and blood with regard to DKD progression. As differentially methylated CpGs at week 9 also emphasized the enrichment of kidney development, this further supports a role of DNA methylation modifications in the blood to renal physiological programming. It is possible that blood and kidney DNA methylation profiles are regulated by the same systemic factors.

In our study, multiple neuronal development processes were commonly enriched in the kidney and blood of animals with advanced DKD, implying a role of the brain–kidney axis in DKD progression. Indeed, sympathetic activation and renal innervation are key players in hypertension and renal pathophysiology [37]. Surgical denervation of the kidney has been shown to attenuate hypertension, albuminuria, TGFβ expression, T cell and macrophage infiltration, as well as interstitial fibrosis in UUO, ischemic, and hypertensive models [38,39]. Chronic bilateral renal denervation also ameliorated glomerular hyperfiltration and renal injury in streptozotocin-induced diabetic rats [40,41], as well as kidney interstitial fibrosis in HFD-fed animals [42]. Although the implication of sympathetic nerves in the pathogenesis of hypertension and chronic kidney disease is well-known, the links between epigenetic regulation, brain–kidney axis, and the progression of DKD have not yet be investigated.

The Wnt signalling pathway was one of the most significant pathways that were enriched in both kidney and blood of animals with advanced DKD at week 32. The Wnt signal cascade is an evolutionarily conserved, developmental pathway that regulates embryogenesis, injury repair, and pathogenesis of human diseases. Mounting evidence has revealed a key role of Wnt signalling in controlling nephrogenesis [43]. Activation of Wnt/β-catenin is instrumental for tubular repair and regeneration after acute kidney injury; however, sustained activation of this signal cascade has been linked to the development of chronic kidney disease and renal fibrosis in unilateral ureteral obstruction (UUO) model [44] and diabetes [45]. The enrichment of Wnt signalling pathway in HFD-fed animals with advanced DKD in our study is consistent with these previous reports, supporting a role of this pathway in chronic kidney diseases.

According to our results, eleven differentially methylated CpGs were detected in the blood of animals with advanced DKD at both early (week 9) and late phases (week 32), suggesting that these abnormal DNA methylations had occurred long before advanced kidney injury developed and persisted across the course of DKD progression. Among these, the most significant CpG corresponds to Fibroblast growth factors (FGF) 22, which was hypermethylated in the rapidly progressing group. Multiple FGFs have been shown to be elevated in patients with chronic kidney disease, including FGF21 and FGF23 [46,47]. The implication of FGF22 has not yet been elucidated; however, mice treated with FGF22 demonstrated reduced pro-apoptosis proteins and increased recovery of spinal cord injury, suggesting tissue protective effects [48].

Among the eleven differentially methylated genes, Angiotensin II receptor (AT1R) type 1b was of particular interest, as AT1R type 1a and 1b are important receptors of angiotensin, playing important roles in the regulation of blood pressure via the renin-angiotensin system (RAS). Renal expression of AT1R increases in diabetic patients with chronic kidney disease and is suppressed by AT1R blockers [21]. Hypomethylation of AT1R type 1a has been correlated with increased AT1R expression in the kidney of mouse offspring exposed to antenatal hypoxia [49]. In our study, AT1R was hypomethylated, which may underline the increased expression of this receptor in diabetic nephropathy. As AT1R is involved in renal innervation and sympathetic activity ^40^, it may also mediate the brain–kidney axis that was enriched in animals with accelerated DKD progression.

## 5. Conclusions

In conclusion, our study identified early differences in blood DNA methylation between animals with Type 2 diabetes that subsequently developed mild or advanced kidney injury. Further studies are required to validate these changes in a larger cohort of animals as well as in diabetic patients with progressive DKD. In addition, gene expression studies are also needed to determine whether these epigenic changes drive kidney-specific transcriptional changes.

## 6. Patents

This section is not mandatory but may be added if there are patents resulting from the work reported in this manuscript.

## Data Availability

The data presented in this study are available on request from the corresponding author.

## Supplementary Materials

The following supporting information can be downloaded at: https://www.mdpi.com/article/10.3390/nu14040785/s1, Figure S1: High-fat diet induced type 2 diabetes in mice; Figure S2: Renal levels of DNA oxidative damage reflected by 8-OHdG staining; Figure S3: Metabolic profiles of HFD-fed animals with and without progressive CKD in comparison to chow; Figure S4: DNA methylation profiling in kidney of HFD-fed mice.
