# Supplementary material for: Blood DNA Methylation Predicts Diabetic Kidney Disease Progression in High Fat Diet-Fed Mice
Source: Nutrients. 2022 Feb 13;14(4):785. doi: 10.3390/nu14040785 (PMC8880442; doi:10.3390/nu14040785)
Supplement: Supplementary file 1 [file nutrients-14-00785-s001.zip › nutrients-1584653-supplementary.pdf]

Blood DNA Methylation Predicts Diabetic Kidney Disease Progression in High Fat Diet-Fed Mice

Long T. Nguyen, Benjamin P. Larkin, Rosy Wang, Alen Faiz, Carol A. Pollock and Sonia Saad

Supplementary document

Methods

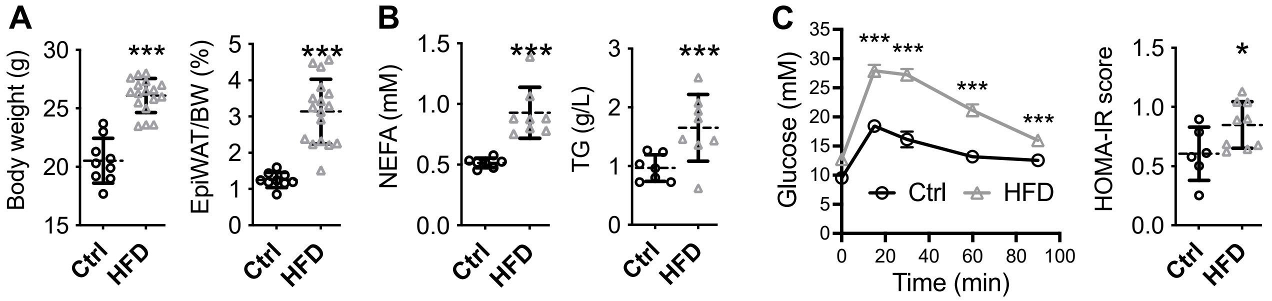

Figure S1. High-fat diet induced type 2 diabetes in mice. (A) increased body weight and fat mass, (B) hyperlipidaemia and (C) insulin resistance. *P<0.05, ***P<0.001.

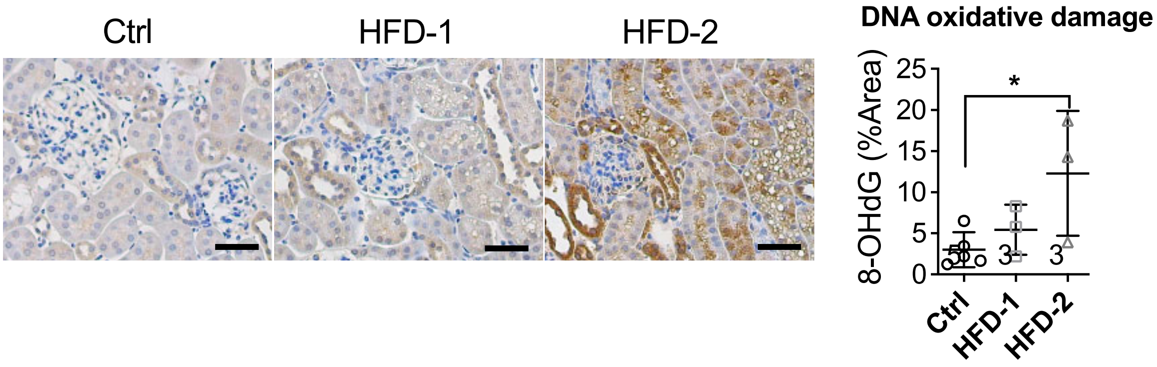

Figure S2. Renal levels of DNA oxidative damage reflected by 8-OHdG staining. *P < 0.05

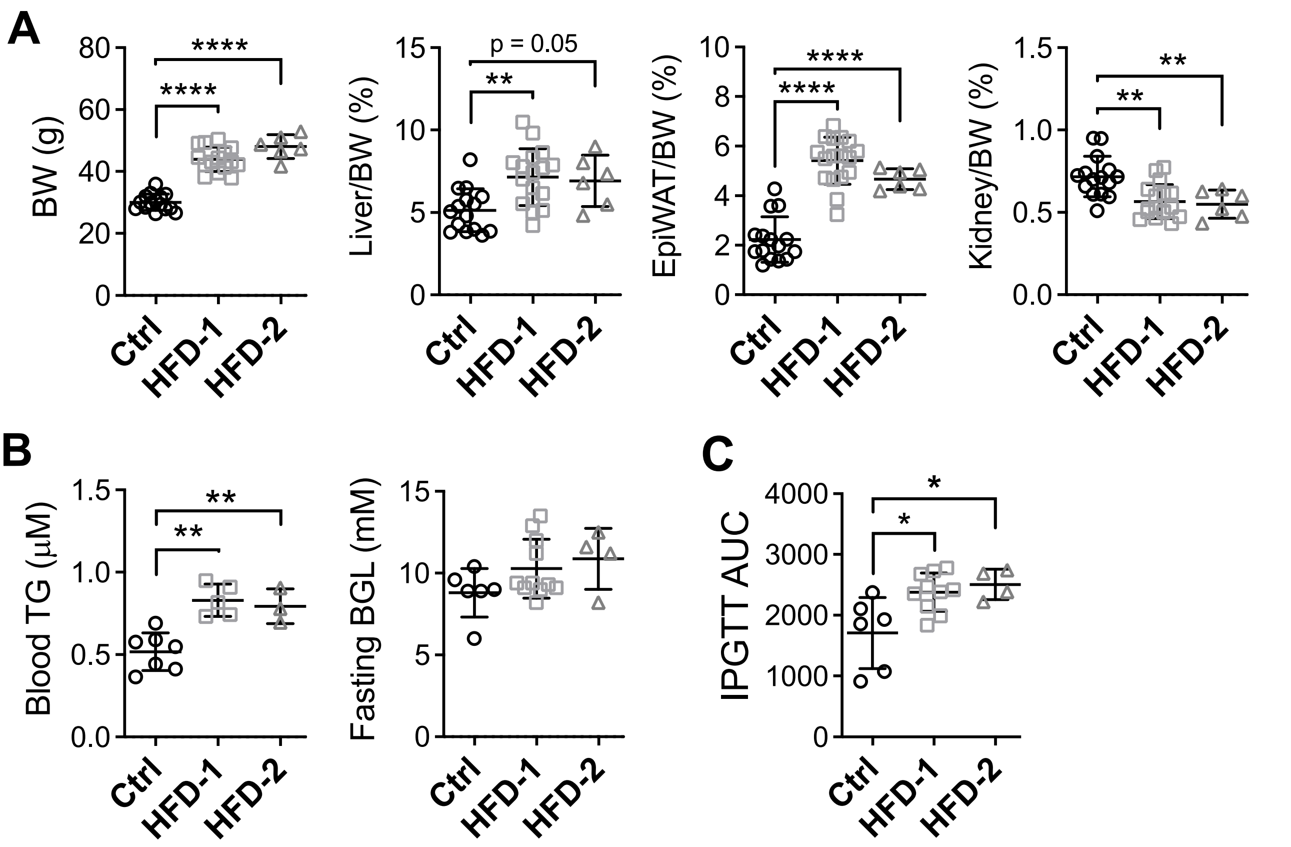

Figure S3. Metabolic profiles of HFD-fed animals with and without progressive CKD in comparison to chow. *P<0.05, **P < 0.01, ****P<0.0001.

A

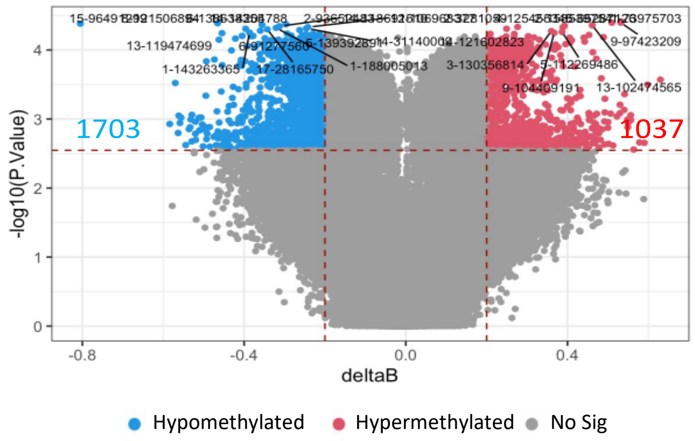

B

Source	Term name	Term ID	P.adj
GO:MF	protein binding	GO:0005515	2.24E-16
GO:MF	binding	GO:0005488	1.07E-09
GO:MF	transcription regulator activity	GO:0140110	3.74E-04
GO:MF	cadherin binding	GO:0045296	8.49E-04
GO:MF	sequence-specific double-stranded DNA binding	GO:1990837	1.28E-03
GO:BP	multicellular organism development	GO:0007275	1.37E-22
GO:BP	anatomical structure development	GO:0048856	3.33E-20
GO:BP	system development	GO:0048731	3.95E-20
GO:BP	neuron differentiation	GO:0030182	4.05E-20
GO:BP	developmental process	GO:0032502	3.38E-19
GO:CC	cell junction	GO:0030054	3.18E-11
GO:CC	glutamatergic synapse	GO:0098978	1.41E-08
GO:CC	cell-cell junction	GO:0005911	8.52E-08
GO:CC	cell projection	GO:0042995	1.02E-07
GO:CC	somatodendritic compartment	GO:0036477	2.36E-07
KEGG	Wnt signaling pathway	KEGG:04310	5.58E-08
KEGG	Glutamatergic synapse	KEGG:04724	8.53E-05
KEGG	Axon guidance	KEGG:04360	1.60E-04
KEGG	Rap1 signaling pathway	KEGG:04015	5.62E-04
KEGG	Melanogenesis	KEGG:04916	9.40E-04
REAC	Neuronal System	REAC:R-MMU-112316	3.77E-06
REAC	Protein-protein interactions at synapses	REAC:R-MMU-6794362	1.01E-04
REAC	Neurexins and neuroligins	REAC:R-MMU-6794361	2.09E-03
REAC	Ca2+ pathway	REAC:R-MMU-4086398	6.68E-03
REAC	Repression of WNT target genes	REAC:R-MMU-4641265	1.11E-02
WP	Wnt Signaling Pathway NetPath	WP:WP539	9.65E-04
WP	TGF Beta Signaling Pathway	WP:WP113	1.40E-02

Figure S4. DNA methylation profiling in kidney of HFD-fed mice. A) Volcano plot of differential methylated loci between advanced (n = 3) and mild (n = 1). The analysis was performed in R using the limma package assuming equal standard deviations. The threshold for changes in methylation level (deltaB) was set at a highly conservative level – 20%. B) Gene-set analysis by G:Profiler. GO: Gene annotation, MF: Molecular function, BP: Biological process, CC: cellular compartment, REAC: Reactome, WP: Wikipathways.
